# The role of R&D and economic policy uncertainty in Sri Lanka’s economic growth

**DOI:** 10.1186/s40854-021-00322-5

**Published:** 2022-01-14

**Authors:** Chandranath Amarasekara, Bernard Njindan Iyke, Paresh Kumar Narayan

**Affiliations:** 1 Central Bank of Sri Lanka, Colombo, Sri Lanka; 2 Monash Business School, Monash University, Melbourne, Australia Wellington Road,; 3 Monash Business School, Monash University, Wellington Road, Melbourne, Australia

**Keywords:** Total factor productivity, Research and development, Endogenous growth theories, Economic policy uncertainty, Sri Lanka, O3, O4

## Abstract

In this paper, we assess the role of investment in research and development (R&D) and economic policy uncertainty (EPU) in Sri Lanka’s economic growth experience. We do this by first determining which endogenous growth theories best explain the evolution of total factor productivity (TFP) in the country. Using historical time series data (1980–2018), we find that semi-endogenous growth theories best explain the evolution of TFP in Sri Lanka. This evidence suggests that R&D is critical to the country’s TFP expansion. We find that, through R&D, EPU has a crucial detrimental impact on TFP growth, although it is short-lived. Our findings are robust and have important implications for R&D investment and for moderating EPU.

## Introduction

Recent events, such as the US–China trade tensions (2018-date),^1^ the recent US presidential election (in 2020), the prolonged Brexit tensions (2016-date),^2^ the persistent political instability in the Middle East, the social unrest in Hong Kong (2019–2020),^3^ the influx of refugees in Europe, the 2020 Russia–Saudi Arabia oil price war,^4^ among others, have heightened global policy uncertainty, which studies argue harms investment and economic growth (see e.g. Jens 2017; Ahir et al. 2018; Phan et al. 2021). The COVID-19 pandemic has further propelled global policy uncertainty to an all-time high and this is estimated to have a severe dampening effect on economic activities across countries (Altig et al. 2020).

Sri Lanka is one of the countries that have experienced and still experience episodes of extreme policy uncertainty. The contributing factors of episodes of extreme uncertainty in Sri Lanka include, among others, the three decades of conflicts in the Northern and Eastern provinces, youth uprisings, the devastating 2004 tsunami, drastic regime shifts, and policy swings (Uyangoda 2010; Lehman 2014). The EPU component of Sri Lanka’s extreme overall uncertainty could be attributed to excessive state budget deficits, drastic regime shifts, high and volatile lending rates, and balance of payments cycles.^5^ These episodes of extreme uncertainty, which have led to a persistently weak business environment, have distorted all forms of investment, trade, and the functioning of the country’s financial market and, consequently, the country’s TFP growth. However, there is little empirical evidence to show the investment and economic growth implications of policy uncertainty for Sri Lanka. Hence, in this paper, we investigate this issue, approaching it from the perspective of endogenous growth theories. Endogenous theories allow us to enrich our empirical exploration with R&D investment and EPU,^6^ while establishing the sources of total factor productivity (TFP) growth in Sri Lanka.

Historical data suggest that Sri Lanka has gradually transitioned from an agriculture-dependent economy towards a service sector-oriented economy with a sizable industrial sector. Public investment in social upliftment—including the provision of universal free education and healthcare—by successive governments, even before independence from the British in 1948, has made Sri Lanka an outlier in terms of human capital development amongst relatively low-income economies for several decades (Osmani 1994). However, Sri Lanka has been unable to convert these investments into a successful economic development story. This is more glaring given that many other economies that were behind Sri Lanka in terms of income levels and human capital development have surpassed Sri Lanka to become industrialised nations. The economic liberalisation programme, which commenced in 1977, resulted in initial gains, but Sri Lanka’s growth performance was severely affected by the bloody internal conflict that raged from 1983 to 2009.^7^ Having recorded an annual growth rate of over 8% for three years, in the immediate aftermath of the conflict, Sri Lanka reverted to an annual growth path of below 5%, which dropped to less than 3% by 2019.^8^

The recent slowdown has been partly attributed to policy uncertainties, which arose from the political instability that prevailed over the past few decades, and the resulting reluctance of domestic as well as foreign investors to expand investment.^9^ While this example gives an indication of how policy uncertainty has dampened investment activities and economic growth in recent times, swings in economic policies observed in Sri Lanka from time to time could provide useful insights into why R&D investment has been lacking in Sri Lanka, despite the availability of a highly educated workforce with a long life expectancy. Motivated by these issues, we aim to assess whether uncertainty, particularly EPU, can explain Sri Lanka’s growth experience via its influence on R&D investment.

Considering the role of policy uncertainty in R&D and economic growth is necessary because growth models, in general, often ignore the role of uncertainty (particularly EPU) in shaping financial markets, R&D investment, and innovation, and, consequently, TFP growth across nations (see e.g. Solow 1956; Romer 1990; Grossman and Helpman 1991). Aside this limitation of theoretical growth models, empirical growth models do not account for policy uncertainty (see Madsen et al. 2010; Ang and Madsen 2011; Juhro et al. 2020). Greater certainty about economic policies would create a conducive environment to investment and, in turn, foster investment of all forms, including R&D investment, consistent with the findings of existing studies. For instance, uncertainty enhances real option values, making investors and firms more cautious when investing or disinvesting (Bloom et al. 2007). Lensink et al. (1999) and Asteriou and Price (2005) find uncertainty has a robust and negative impact on economic growth. Rigotti and Shannon (2005) demonstrate that, in a general equilibrium model, some assets are not traded under uncertainty, because uncertainty leads to incomplete markets (Vorbrink 2014). Policy uncertainty can generate instability in the financial market, weakening equity prices and exposing firms to bankruptcy. Belke et al. (2018) demonstrate that Brexit-induced policy uncertainty caused instability in the British and other linked financial markets. Uncertainty can also reduce the pace of TFP growth by impeding trade flows (Baum and Caglayan 2010). Alessandria et al. (2015) demonstrate that uncertainty shocks can induce the international reallocation of production. He et al. (2020) find that low (high) EPU is positively (negatively) associated with corporate innovation.

Our study addresses these limitations of the literature by showing that semi-endogenous growth theories best explain the growth experience of Sri Lanka, such that R&D investment is a fundamental driver of technological growth and should be intensified for sustained economic growth. In addition, we show that EPU impedes technological progress, consistent with theoretical predictions. It is interesting to note that the negative impact of EPU dissipates quickly. We show that, despite the negative impact of EPU, R&D investment recovers after one year following the negative uncertainty shock, which spurs TFP growth. This finding suggests that the Sri Lankan economy internalised the EPU shocks after having gone through frequent, prolonged periods of uncertainty.

Our study contributes to three strands of literature. First, it contributes to the endogenous growth and R&D literature by constructing and testing semi-endogenous and Schumpeterian growth theories for Sri Lanka. This is important because the literature is presently undecided regarding whether the Schumpeterian growth or semi-endogenous theories best explain the growth experience of developing countries like Sri Lanka (see Ha and Howitt 2007; Madsen 2008). The Sri Lankan specific studies, such as the works of Dutz and O’Connell (2013), Kumari and Tang (2019), and Nugawela (2019), do not discriminate between these models leaving policymakers uncertain regarding how to adjust the non-traditional TFP factors (R&D, human capital, trade, etc.) to achieve sustainable growth. Thus, our exploits establish the most appropriate growth framework for understanding R&D and growth dynamics in Sri Lanka. To our knowledge, our study is the first to cover the Sri Lankan case. Understanding what growth model best explains the country’s growth experience is a step forward to identifying the most important growth determinants and thus provides guidance for more focused growth policies.

Second, uncertainty is an important disruptor of economic activity, as established in various studies (Lensink et al. 1999; Asteriou and Price 2005; Rigotti and Shannon 2005; Bloom et al. 2007; Baum and Caglayan 2010; Alessandria et al. 2015; Belke et al. 2018). However, endogenous growth theories remain silent on the role of uncertainty in TFP growth dynamics. This silence is maintained by Sri Lankan specific studies like the works of Dutz and O’Connell (2013), Kumari and Tang (2019), and Nugawela (2019), which only mentioned the potential effect of the armed conflict on TFP growth. The lack of research on this issue is surprising, given the country’s frequent episodes of uncertainty. We extend the uncertainty literature by demonstrating that policy uncertainty is an important determinant of TFP growth.

Third, nonlinearity between TFP growth and its determinants is an important but often ignored feature of growth models (Juhro et al. 2020). All countries face structural changes from time to time. In the Sri Lankan context, sudden changes in political regimes are common, in addition to conflicts and civil unrests (see Uyangoda 2010; Lehman 2014). These sudden changes are often associated with a sharp decline in macroeconomic variables and can change the relationship between TFP growth and its determinants over time. Such a nonlinear relationship has been acknowledged by prior studies (see e.g. Madsen et al. 2010). Our study considers potential nonlinearity in empirical tests. We depart from prior studies by endogenously modelling the nonlinearity between TFP growth and R&D and policy uncertainty.^10^

We proceed as follows. Section 2 motivates endogenous growth models for Sri Lanka. Section 3 specifies our endogenous growth models. Section 4 presents the data. Section 5 reports and discusses the results, and Sect. 6 concludes the paper.

## Motivating endogenous growth models for Sri Lanka

Neoclassical growth theories emphasize the role of exogenous TFP in driving growth in the long run (see Solow 1956). However, endogenous growth theories argue that TFP is endogenous and hence countries can raise long-run growth by raising TFP determinants (Romer 1990; Grossman and Helpman 1991; Aghion and Howitt 1992). In this section, inspired by Juhro et al. (2020), we motivate the case for endogenous growth models in Sri Lanka using historical data.

Our motivation is grounded in a neoclassical growth accounting exercise, following the seminal work of Solow (1956). In the neoclassical growth model, growth is a function of labour, capital, and exogenous technology. The textbook model is formulated as:

1 $$y = Ak^{\alpha }$$

where $y = Y/L$ is the output per capita, $k = K/L$ is the capital per capita, $A$ is TFP, and $\alpha$ is the capital share in the output. If we log-linearise and differentiate Eq. (1) with respect to time, we derive growth, $g$, which depends on TFP growth ($\Delta A$) and capital per capita growth ($\Delta k)$ with respect to time. This can be written as

2 $${\Delta }y = {\Delta }A + \alpha {\Delta }k$$

TFP ($A$) and the capital share in the output ($\alpha$) are not observable and must be inferred from labour, capital, and output, which are observable. We can assume that $\alpha$ is the capital share in national income, following Aghion and Howitt (2009, p. 106), and rewrite Eq. (2) as

3 $${\Delta }A = {\Delta }y - \alpha {\Delta }k$$

We extract historical data (1960–2017) on the real gross domestic product GDP ($Y$), employment ($L$) and one minus the labour compensation share in the GDP ($\alpha$) (i.e. $1{-}LABSH = \alpha$) from the Penn World Table 9.0 database, to carry out a basic growth accounting exercise for Sri Lanka.^11^ We estimate a mean output per capita growth ($\Delta y$) of 3.46%, a mean TFP growth ($\Delta A$) of 2.78%, and a mean capital per capita growth ($\Delta k$) of 2.64% for this sample. It is evident that TFP growth contributes significantly to output per capita growth, just as capital per capita. The picture is even clearer when we look at the shares in economic growth. The shares of TFP and capital in economic growth are 0.80 and 0.20, respectively.

**Fig. 1:**
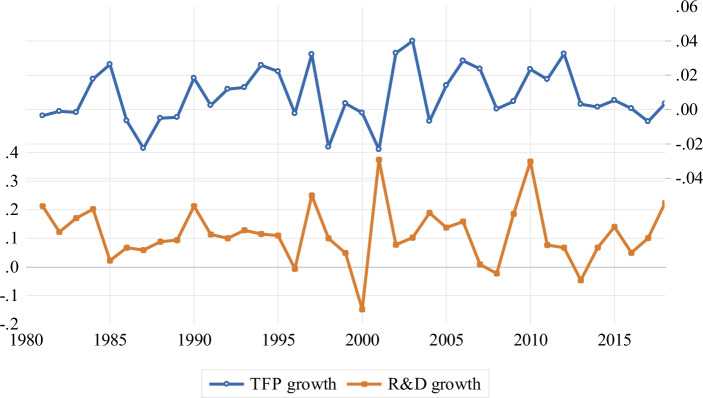
Sri Lankan TFP and R&D growth from 1980 to 2018. The figure depicts the evolution of TFP and R&D growth in Sri Lanka from 1980 to 2018. The growth in these variables are measured in terms of the first differences of their logarithm. The TFP data is from the Federal Reserve Economic Data (FRED), while the R&D data is from the World Development Indicators (WDI) and the National Science Foundation (NSF)

Our basic growth accounting exercise indicates that TFP growth is a primary determinant of Sri Lanka’s economic growth. Hence, naturally, policymakers and researchers seek to understand factors driving TFP growth. Endogenous growth theories stress the importance of R&D or innovation in driving TFP growth (Juhro et al. 2020). As a preliminary test of this contention, we appeal to the evolution of TFP and R&D growth in Sri Lanka from 1980 to 2018. This is the longest period over which some clear inference on these two indicators can be made. Figure 1 shows that the evolution of TFP growth looks very much like the growth of R&D, indicating that the growth of investment in R&D, despite its low level, is an important driver of TFP in Sri Lanka.

Since R&D is an important determinant of TFP growth, any disruptor of R&D investment would undermine TFP growth. The real options literature explains that firms are generally cautious during periods of uncertainty (Bloom et al. 2007). Since the decision to invest depends on the net present value of the project, which, in turn, depends on parameters of the economy—uncertainty being the most important—investors must act cautiously when facing uncertainty, particularly regarding long-term investment (Sarkar 2000; Iyke and Ho 2020). At the national level, governments’ decisions on R&D are consistent with those at the firm level, although the latter might be less concerned about the social benefits of R&D investments; in other words, governments and policymakers are also expected to be cautious under uncertainty. Hence, the level of R&D investment in a country is contingent on uncertainty. We build on the possible disruptive channels of uncertainty on TFP growth by introducing and testing endogenous growth theories for Sri Lanka. We test the hypothesis that uncertainty (in this case EPU) impedes TFP growth through R&D.

## Specification of endogenous growth models

Having established in Sect. 2 that TFP is a critical driver of Sri Lanka’s growth experience, we now outline the main endogenous growth theories explaining the sources of TFP growth, namely, Schumpeterian growth and semi-endogenous theories. These theories are similar, in that they both argue that R&D or innovation is the primary determinant of TFP growth. Their main difference is that, whereas Schumpeterian growth theories assume constant returns to knowledge and the increasing complexity of new innovations, semi-endogenous growth theories relax the assumption of constant returns to knowledge (see Madsen 2008). According to Ha and Howitt (2007), Madsen (2008), and Juhro et al. (2020), the following discrete time log-linearised idea production function is used to differentiate endogenous growth theories:

4 $$\Delta lnA_{t} = \ln \lambda + \sigma \left[ {lnX_{t} - lnQ_{t} + \left( {\frac{\phi - 1}{\sigma }} \right)lnA_{t} } \right]$$

where $Q \propto L^{\beta }$ is in steady state, and $\Delta lnA$, $A$, $X$, $Q$, and $X/Q$, denote, respectively, TFP growth, the TFP level, research inputs, product variety, and research intensity. Prior studies (e.g. Zachariadis 2003; Griffith et al. 2004; Madsen 2008) proxy for product variety with real output ($Y$), labour ($L$), and the product of TFP and labour ($AL$). The parameters $\lambda$, $\sigma$, $\phi$, and $\beta$ denote, respectively, R&D productivity, duplication, returns to scale in the knowledge function, and product proliferation parameters. Duplication ($\sigma$) takes the value of one if none of the new innovations are imitations, and zero if all innovations are imitations of prevailing knowledge.

Equation (4) implies that $lnA$ is nonstationary, so that $\Delta lnA$ stationary. We can test this condition using unit root tests. Alternatively, we can present this equation piece by piece to test the growth theories, as follows:

5 $$\nu_{t} = lnX_{t} + \left( {\frac{\phi - 1}{\sigma }} \right)lnA_{t} { }$$

6 $$\varsigma_{t} = lnX_{t} - lnQ_{t}$$

Using Eq. (5), semi-endogenous growth theory is valid if $lnX$ and $lnA$ are cointegrated and the cointegrating vector is ($1, \left( {\phi - 1} \right)/\sigma$), such that the second element carries a negative sign, indicating diminishing returns to knowledge ($\phi < 1$). Using Eq. (6), the Schumpeterian growth model is valid if $lnX$ and $lnQ$ are cointegrated, with the cointegrating vector being (1, − 1). These are stricter conditions that are rarely observed in practice. Hence, the evidence of cointegration is adequate for concluding in favour of either model (Juhro et al. 2020).

Equations (5) and (6) are only suitable for discriminating endogenous growth theories (see Madsen 2008). To examine the impact of EPU on TFP growth, we extend the empirical models of prior studies (see Madsen et al. 2010; Juhro et al. 2020) by regressing TFP growth on EPU and a range of determinants. This regression is as follows:

7 $$\begin{aligned} \Delta lnA_{t} & = \gamma_{0} + \gamma_{1} (\Delta \ln X_{t}^{d} )*(\Delta EPU_{t} ) + \gamma_{2} (\Delta \ln X_{t - 1}^{d} )*\left( {\Delta EPU_{t - 1} } \right) \\ & \quad + \gamma_{3} (\Delta \ln X_{t - 2}^{d} )*\left( {\Delta EPU_{t - 2} } \right) + \gamma_{4} (\Delta \ln X_{t - 3}^{d} )*\left( {\Delta EPU_{t - 3} } \right) + \gamma_{5} \Delta lnX_{t}^{f} \\ & \quad + \gamma_{6} ln\left( \frac{X}{Q} \right)_{t}^{d} + \gamma_{7} ln\left( \frac{X}{Q} \right)_{t}^{f} + \gamma_{8} ln\left( {\frac{A}{A}_{LKA}^{JPN} } \right)_{t - 1} + e_{t} \\ \end{aligned}$$

where $X$, $A$, $Q$, and $A^{JPN} /A^{LKA}$ denote, respectively, R&D expenditures, TFP, product variety, and the distance to the technology frontier.^12^ The superscripts $d$ and $f$ indicate domestic and foreign, respectively, while $JPN$, $LKA$, $\gamma_{i}$, and $e$ denote, respectively, Japan, Sri Lanka, the parameters, and a stochastic error term.

We examine our hypothesis that EPU influences TFP growth via the R&D by interacting domestic R&D, $X^{d}$, with domestic EPU. Our motivation for modelling R&D and EPU in this way is as follows. Basu et al. (2006) argue that an enhancement in technology contributes to a decline in inputs and investments in the short run; therefore, output is expected to decline. We therefore argue that, if technological progress eventuates in a period marked by EPU, the effect on output could be severe. Another line of argument is owed to Hsu (2009), who contends that, while technologies contribute to greater productivity, they also raise economic uncertainty. The point is that if technological progress takes place at a time the economy is undergoing a phase of EPU, the economic uncertainty will multiply, and the effects of productivity might not be as healthy as under normal circumstances. Sudden changes in governments would have a lasting impact on investment in R&D and in turn TFP growth. Thus, the lag R&D and EPU interaction terms capture the persistency of the impact of these variables on TFP growth.

## Data

We collected data from several sources for our analysis, including the Central Bank of Sri Lanka, the IMF’s World Economic Outlook, the World Development Indicators, the National Science Foundation of Sri Lanka, the World Intellectual Property Organization, the Penn World Tables 9.0, and the Federal Reserve Economic Data. Table A.1 in the “Appendix” provides the definitions/full names of the variables and their sources.

**Table 1:** Stationarity tests

Perron (1989) test				
Variable	*t*-statistic	TB1	Lags	Status
*lnA*	− 2.332	2001	0	I(1)
*lnRD*	− 1.861	2000	0	I(1)
*lnPATENT*	− 5.124	1994	1	I(0)
*lnRD/L*	− 1.731	2000	0	I(1)
*lnRD/Y*	− 2.603	2000	0	I(1)
*lnRD/AL*	− 2.053	2000	2	I(1)
*lnPATENT/L*	− 4.819	1994	1	I(0)
*lnPATENT/Y*	− 4.598	1994	2	I(0)
*lnPATENT/AL*	− 5.162	1994	1	I(0)

Variable	Narayan–Popp (2010) test									
	M1						M2			
	Test statistic	TB1	TB2	k	Status	Test statistic	TB1	TB2	k	Status
*lnA*	− 3.119	1996	2000	0	I(1)	− 1.709	1996	2001	0	I(1)
*lnRD*	− 5.031	1999	2009	0	I(0)	− 4.192	1999	2009	0	I(1)
*lnPATENT*	− 5.630	1993	1998	2	I(0)	− 4.040	1993	1996	2	I(1)
*lnRD/L*	− 4.314	1999	2009	0	I(0)	− 3.314	1999	2009	0	I(1)
*lnRD/Y*	− 5.708	1999	2009	0	I(0)	− 4.617	1999	2009	0	I(1)
*lnRD/AL*	− 2.970	2000	2009	0	I(1)	− 4.162	1999	2009	0	I(1)
*lnPATENT/L*	− 4.075	1993	2002	1	I(1)	− 4.564	1994	2002	2	I(1)
*lnPATENT/Y*	− 5.287	1993	1998	2	I(0)	− 3.775	1993	2002	2	I(1)
*lnPATENT/AL*	− 3.247	1994	1996	2	I(1)	− 1.664	1994	2002	2	I(1)

The table shows the results of the stationarity tests. We compared the Perron (1989) *t*-statistic, and Narayan and Popp (2010) M1 and M2 statistics to the critical values reported in their respective studies. We selected the lags in the Perron test automatically using the Schwarz information criterion (SIC). The lags in the Narayan and Popp (2010) test are chosen using Hall’s (1994) procedure. Perron’s (1989) test accommodates a single structural break, while Narayan and Popp’s (2010) test accommodates two structural breaks. Only the intercept term is included in the test regressions. k, TB1, and TB2 are the optimal lags, the first, and second structural break dates, respectively. Our sample is from 1980 to 2018

For some variables, observations are missing for certain years. For example, R&D expenditure data are missing in 2005, 2007, 2009, 2011–2012, 2016–2018, and 1980–1995, and labour force data are missing in 1980, 1983–1984, and 1987–1989. We handle these missing observations as follows. If data are missing between two adjacent observations, then we replace the missing observation by the average of the adjacent observations. Similarly, if data are missing within a neighbourhood, then we replace the missing observation by the average of the two successive observations. If observations are missing over an extended period (e.g. 1980–1995), we backcast and forecast the missing observations using an exponential growth curve.^13^

We follow the literature and use labour and real output to measure product variety (see e.g., Madsen et al. 2010; Juhro et al. 2020). We measure real output (*Y*) and labour (*L*) as the GDP at constant 2010 national prices and the number (in millions) of persons engaged, respectively. We measure $A$ as the TFP at constant national prices (with the year 2011 corresponding to 100). Our measures of R&D input or innovation ($X$) are government R&D expenditures ($RD$) and the number of patent applications by domestic residents ($PATENT$). Data on Sri Lanka’s government R&D expenditures are not available. Hence, we estimate these as the product of R&D expenditures as the percentage of the GDP (RDGDP) and the nominal GDP scaled by 100 (i.e. (RDGDP*GDP*100)/100). Following prior studies (e.g. Madsen et al. 2010; Juhro et al. 2020), we measure research intensity ($X/Q$) as $RD/Y$, $RD/L,$ $RD/AL$, $PATENT/Y$, $PATENT/L$, and $PATENT/AL$. We adjust $RD$ and $PATENT$ by TFP (i.e. $RD/AL$ and $PATENT/AL$) to capture the growing complexity of new innovations that results from economic advancement (Aghion and Howitt 1992; Madsen et al. 2010). We measure EPU using the news-based World Uncertainty Index for Sri Lanka developed by Ahir et al. (2018).^14^ This news-based measure of EPU is derived from the frequency of the word *uncertainty* and its variants in Sri Lanka’s quarterly Economist Intelligence Unit reports (Ahir et al. 2018). The authors show that the index is associated with higher EPU and lower income growth, risk, and stock market volatility.

**Fig. 2:**
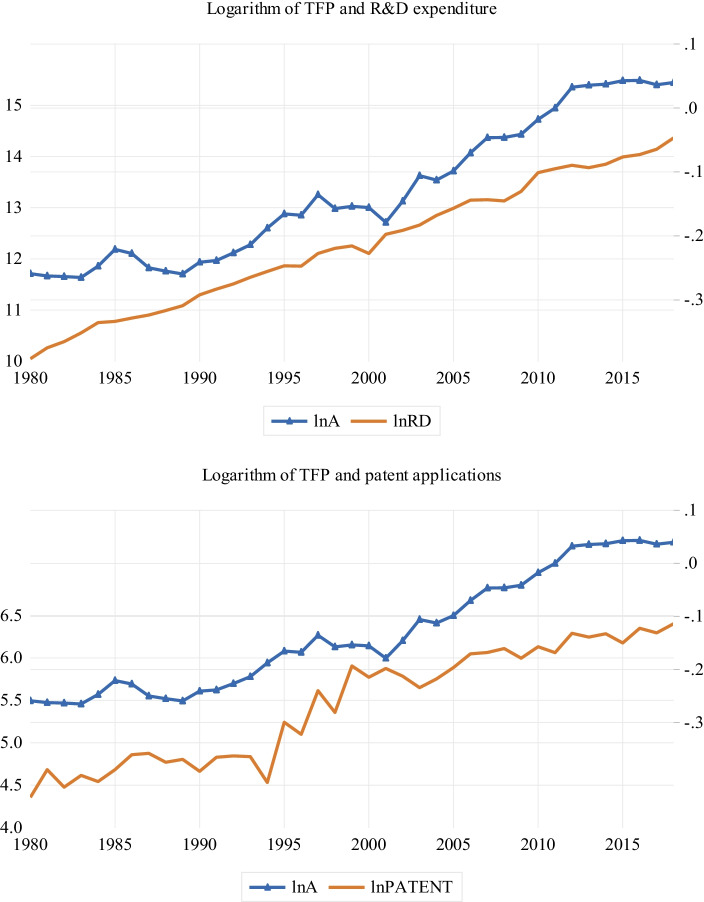
TFP and R&D trends. The figure shows that movements of TFP and R&D indicators over time. $lnA$, $lnRD$, and $lnPATENT$ denote, respectively, the logarithms of TFP, real R&D expenditure, and patent applications from residents. The sample period is from 1980 to 2018

Figure 2 shows the trends in TFP and R&D measures (i.e. R&D expenditures and patent applications) from 1980 to 2018. The graph shows that R&D investment in Sri Lanka increased over time, and such investment led to the rise in TFP. It is obvious that R&D expenditures have been an important driver of TFP in the country. For example, R&D expenditure substantially declined between 1999 and 2000, which caused a significant decline in TFP even after 2000. We see a similar pattern between TFP and patent applications in the bottom panel of Fig. 2. The rising number of patent applications is accompanied by rising TFP. Similar to the decline in R&D expenditures during 1999–2000, patent applications also declined during this period, which was followed by a decline in TFP. In short, there is clear positive relation between TFP and R&D activity/innovation, as shown in Fig. 2.

**Fig. 3:**
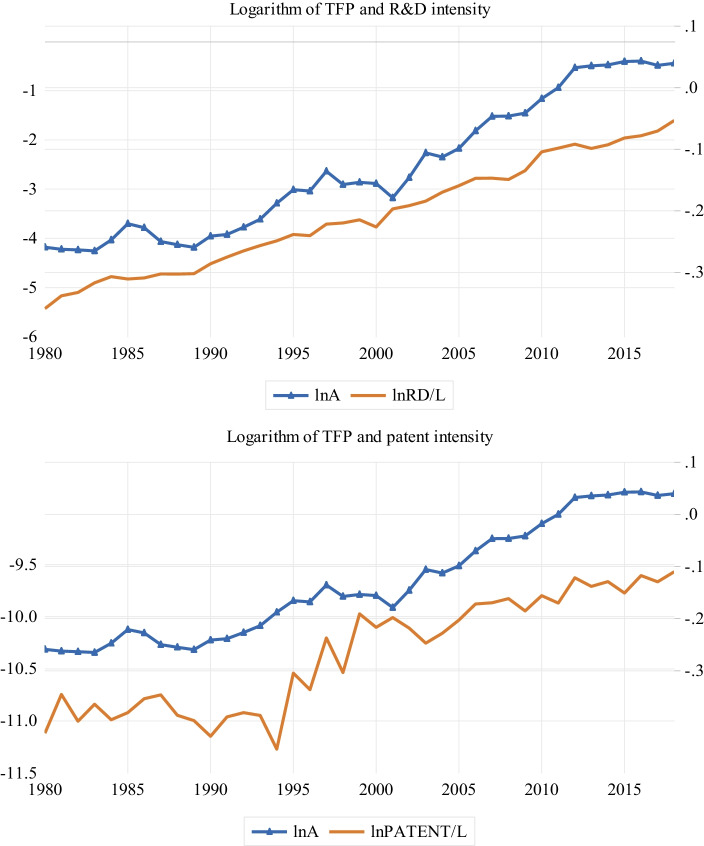
TFP and R&D intensity trends. The figure shows the movements of TFP and R&D intensity indicators over time. $lnA$, $lnRD/L$, and $lnPATENT/L$ denote, respectively, the logarithms of TFP, real R&D intensity, and patent intensity. The sample period is from 1980 to 2018

Figure 3 is the equivalent graph for the TFP and R&D intensity measures (i.e. R&D and patent intensities). We observe similar patterns as in Fig. 2; that is, the R&D intensity measures are positively related to TFP. Most importantly, there is a clear upward trajectory in the R&D intensity measures, and it is associated with rising TFP.

Figures 2 and 3 suggest that both growth theories (i.e. the Schumpeterian growth and semi-endogenous theories) are supported by the data. Figure 2 shows that TFP growth in Sri Lanka is driven by innovation, while Fig. 3 shows that TFP growth is driven by innovation intensity. Theoretically, the data do not always support both growth models. For example, Madsen (2008) finds support for Schumpeterian growth models for 21 Organisation for Economic Co-operation and Development countries, but no support for semi-endogenous growth models. Similarly, Ang and Madsen (2011) find limited evidence in favour of semi-endogenous growth models and strong support for Schumpeterian growth models in China, India, Japan, Korea, Singapore, and Taiwan over the period from 1953 to 2006. In contrast, Juhro et al. (2020) find it difficult to distinguish semi-endogenous growth models from Schumpeterian growth models in Indonesia, but the evidence appears to favour semi-endogenous growth models. The graphs are consistent with the finding of Barcenilla-Visús et al. (2014), that both theories hold true in Canada, Finland, France, Italy, Spain, and the United States from 1979 to 2001. It is important to note that the positive association observed between TFP and innovation (intensity) could be nonsignificant when subjected to rigorous testing. Hence, it what follows, we seek to verify the positive association based on the intuitions outlined in Sect. 3.

## Results

### Test for stationarity

Recall that an implication of Eq. (4) is that semi-endogenous models hold true if TFP ($lnA$) is a unit root [I(1)] process, so that, by extension, the R&D and patent variables ($lnRD$ and $lnPATENT$) are I(1) processes. Similarly, Eq. (4) implies that Schumpeterian models hold if the R&D intensity measures (i.e. $RD/Y$, $RD/L$ $RD/AL$, $PATENT/Y$, $PATENT/L$, and $PATENT/AL$) and TFP ($lnA$) are I(0), or stationary, processes (Ang and Madsen 2011). We can test these implications using unit root tests. Figures 2 and 3 show that the variables do not evolve along a smooth path and are hence best tested using unit root tests, which accommodate this behaviour. We draw on two structural break unit root tests, namely, the tests of Perron (1989) and Narayan and Popp (2010, NP), to examine the unit root properties of the variables. The Perron and NP tests account for one and two structural breaks, respectively.

Table 1 shows the test results. The Perron test fails to reject the null hypothesis of no unit root for $lnA$. This finding is supported by the NP test results, which means that TFP is nonstationary, consistent with semi-endogenous growth theory. R&D expenditures are also nonstationary, based on both tests. However, the evidence of unit roots in patent applications is unclear, because, whereas the Perron test finds the variable to be stationary, the NP test suggests mixed conclusions. In other words, the M1 statistics are consistent with the Perron test, but the M2 test is not. With regards to the R&D intensity measures, the Perron test suggests that $RD/Y$, $RD/L$, and $RD/AL$ are nonstationary, whereas $PATENT/Y$, $PATENT/L$, $PATENT/L$, and $PATENT/AL$ are. The NP test results suggest mixed conclusions by appearing to support the nonstationarity of the R&D intensity measures. Overall, both tests appear to support the semi-endogenous growth models.

### Cointegration results

The unit root tests in the preceding section (i.e. Sect. 5.1), which also double as tests for structural breaks, indicate that the variables are subjected to structural breaks. This finding is also supported by the evolution of the variables as depicted in Figs. 2 and 3.^15^ Aside from this, TFP and the R&D indicators appear to co-move over time. This finding demands formal nonlinear testing to establish a long-run relation between TFP and the R&D indicators. In addition, Eq. (5) suggests that semi-endogenous growth theory is valid if $lnX$ and $lnA$ are cointegrated and the cointegrating vector is ($1, \left( {\phi - 1} \right)/\sigma$), such that the second element carries a negative sign. Similarly, Eq. (6) suggests that Schumpeterian growth theory is valid if $lnX$ and $lnQ$ are cointegrated, with the cointegrating vector being (1, − 1). However, as argued in Sect. 3, these are stricter conditions, which are rarely observed in practice. Thus, following Juhro et al. (2020), we find adequate cointegration evidence to conclude in favour of either model.

We apply two nonlinear cointegration tests to verify that TFP and the R&D indicators share common long-run relationships. Specifically, we apply the Gregory–Hansen and nonlinear autoregressive distributed lag (NARDL) bounds tests. The Gregory–Hansen test has a null hypothesis of no cointegration, which is tested against the alternative of cointegration with regime shifts. The NARDL test has the null hypothesis of no cointegration against an alternative of cointegration. The main intuition underlying this test is that negative and positive changes in a predictor have a nonlinear impact on the predictand. Table 2 reports the results of these tests. Both tests show strong evidence of cointegration between TFP and the R&D indicators at the conventional levels of statistical significance. In other words, the cointegration tests support both growth models, such that the necessary conditions for semi-endogenous growth and Schumpeterian theories are satisfied within the nonlinear cointegration framework.

**Table 2:** Cointegration tests

Equation	Panel A: Gregory-Hansen test			
	ADF (*t*-statistic)	Phillips (Zt-statistic)	Lag	Break
*lnX* = *f(lnY, lnA)*	− 4.960**	− 5.027*	0	2002
*lnX* = *f(lnAL, lnA)*	− 5.405**	− 5.309**	0	1999

Equation	Panel B: NARDL bounds test				
	Test statistic	Value	Significance	I(0)	I(1)
*lnX* = *f(lnY, lnA)*	F-statistic	6.330***	10%	2.460	3.460
	k	2	5%	2.947	4.088
			1%	4.093	5.532
*lnX* = *f(lnAL, lnA)*	F-statistic	10.170***			
	k	2			

The table shows the cointegration test results. In Panel A, we report the Gregory-Hansen test results. This test has a null hypothesis of no cointegration, which is tested against the alternative of cointegration with regime shifts. The optimal lag for this test is based on the Schwarz information criterion. We find that the regime shift occurred either in 1999 or 2002. In Panel B, we report the nonlinear autoregressive distributed lag (NARDL) bounds test results. The null hypothesis under this test is that there is no cointegration. The main intuition underlying this test is that negative and positive changes in a predictor has nonlinear impact on the predictand. k, I(0), and I(1) denote, respectively, the number of predictors in the equation, and the critical values for the lower and upper bounds. In both tests, we included a maximum of 2 lags in the test regressions. To test the semi-endogenous growth theory, we estimate $\nu_{t} = lnX_{t} + \left( {\left( {\phi - 1} \right)/\sigma } \right)lnA_{t}$, where $A$ is TFP and $X$ is R&D expenditure. Similarly, to test the Schumpeterian growth theory, we estimate $\varsigma_{t} = lnX_{t} - lnQ_{t}$, where $Q$ denotes product quality (i.e. $Y$ or $AL$), and $ln$ denotes the natural logarithm

*, **, and ***Statistical significance at 10%, 5%, and 1% levels, respectively.

### Long-run elasticities

Equations (5) and (6) imply that $lnX_{t} = \mu lnQ_{t} + \kappa lnA_{t} + e_{t}$, where $\kappa = \left( {1 - \phi } \right)/\sigma$ (Zachariadis 2003, 2004). Within this framework, semi-endogenous growth theory is valid if $\kappa > 0$ and $\mu = 0$, and $e_{t}$ is a stationary error term. Similarly, the Schumpeterian theory hypothesis holds if $\kappa = 0$ and $\mu = 1$. This means that we can verify the validity of both theories by estimating $\mu$ and $\kappa$, the product variety and technical elasticities of R&D/innovation.

Because the endogenous growth theories imply a long-run relation between TFP and innovation, the most appropriate way of estimating the elasticities should account for this long-run property. To this end, we draw on the dynamic least squares approach when estimating the elasticities. Table 3 reports these results. We report the results of the fully specified regression and the piecewise regressions (i.e. Eqs. (5) and (6)) in Panels A and B, respectively. The results suggest that TFP and product variety are critical determinants of R&D expenditure. This finding refutes the joint hypothesis under the semi-endogenous and Schumpeterian growth theories. None of the conditions is satisfied if product variety is measured as $Y$ (i.e. $\kappa < 0$ and $\mu > 0$), while semi-endogenous growth theory is partly satisfied if product variety is measured as $AL$ (i.e. $\kappa > 0$ and $\mu > 0$). Considering the piecewise regressions in Panel B, none of the Schumpeterian conditions (i.e. $\kappa = 0$ and $\mu = 1$) is satisfied but the semi-endogenous conditions ($\kappa > 0$ and $\mu = 0$) are partly satisfied; that is, we cannot reject the condition that $\kappa > 0$. Hence, the evidence seems to favour semi-endogenous growth theory. This evidence is consistent with that documented by Juhro et al. (2020) for Indonesia.

**Table 3:** Long-run elasticities

Panel A: Long-run elasticities			
Variable	Coefficient (*p* value)	Variable	Coefficient (*p* value)
*lnY*	2.388*** (0.000)	*lnAL*	2.481*** (0.000)
*lnA*	− 1.685* (0.064)	*lnA*	5.492*** (0.000)
Constant	− 7.571*** (0.000)	Constant	− 25.882*** (0.000)
Adjusted R^2^	0.995	Adjusted R^2^	0.993

Panel B: Piecewise long-run elasticities			
Variable	Coefficient (*p* value)		
*lnA*	10.755*** (0.000)	–	–
*lnY*	–	2.086*** (0.000)	–
*lnAL*	–	–	4.886*** (0.000)
Constant	13.676*** (0.000)	− 4.926*** (0.000)	− 64.324*** (0.000)
Adjusted R^2^	0.956	0.991	0.953

The table shows the long-run elasticity estimates obtained using the dynamic ordinary least squares (DOLS) approach. Panel A estimates the specification $lnX_{t} = \mu lnQ_{t} + \kappa lnA_{t} + e_{t}$, where $\kappa = \left( {1 - \phi } \right)/\sigma$. Schumpeterian growth theory holds true if $\kappa = 0$ and $\mu = 1$, while semi-endogenous growth theory holds true if $\kappa > 0$ and $\mu = 0$. $X$, $A$*,* and $Q$, denote, respectively, R&D expenditure, TFP, product quality (i.e. $Y$ or $AL$). Panel B estimates the piecewise regressions $\nu_{t} = lnX_{t} + \left( {\left( {\phi - 1} \right)/\sigma } \right)lnA_{t}$ and $\varsigma_{t} = lnX_{t} - lnQ_{t}$. $lnX_{t}$ is the dependent variable. In the Schumpeterian growth model, the predictor is either $lnY$ or $lnAL$, whereas in the semi-endogenous growth model, it is $lnA$. We used fixed lags and leads of one and estimate the Newey-West fixed bandwidth based long-run variance. * and *** indicate, respectively, statistical significance at 10% and 1% levels. Coefficients and *p-value*s are, respectively, outside and inside the parentheses. Our sample is from 1980 to 2018

### The impact of EPU on TFP growth

Having established that Sri Lanka’s growth is best explained by semi-endogenous growth theory, we now turn to testing our hypothesis that EPU determines the impact of R&D investment on TFP growth. The usual way of completing the test of endogenous growth theories is to regress TFP growth on R&D, R&D intensity, and technology frontier indicators (Juhro et al. 2020). However, such a regression would ignore the role of EPU in TFP growth. Hence, we extend the regression to capture the impact of EPU, proxied by the news-based world uncertainty index for Sri Lanka constructed by Ahir et al. (2018), by interacting the R&D indicator with an EPU indicator. As discussed earlier, policy uncertainty has been persistent in Sri Lanka. We accommodate policy uncertainty persistence by including three lags of the interaction term in our model.

We report estimates of our model, that is, Eq. (7), in Table 4. In Column (1), we include only the contemporaneous interaction term, while, in Column (4), we include the contemporaneous interaction term and its lagged terms of up to three. The estimates suggest that EPU reduces TFP growth via its impact on R&D, as indicated by the coefficient of the contemporaneous interaction term, which is negative and statistically significant. Over time, the economy adjusts to the EPU shock, such that its impact on TFP growth via R&D is no longer negative but positive, as shown by the coefficients of the lagged interaction terms, which are positive and significant up to the second lag. By the third year (third lag), the economy becomes immune to EPU, as shown by the coefficient of the third lagged interaction term, which is positive but statistically nonsignificant.

**Table 4:** Impact of EPU on TFP growth

	(1)	(2)	(3)	(4)
Constant	− 0.201** (0.034)	− 0.129 (0.243)	− 0.016 (0.886)	0.057 (0.666)
$\left( {\Delta lnX_{t}^{d} } \right)*\left( {\Delta EPU_{t} } \right)$	− 0.448** (0.010)	− 0.383** (0.023)	− 0.322** (0.030)	− 0.327** (0.041)
$\left( {\Delta lnX_{t - 1}^{d} } \right)*\left( {\Delta EPU_{t - 1} } \right)$		0.285** (0.037)	0.414*** (0.002)	0.436*** (0.002)
$\left( {\Delta lnX_{t - 2}^{d} } \right)*\left( {\Delta EPU_{t - 2} } \right)$			0.386*** (0.002)	0.401*** (0.002)
$\left( {\Delta lnX_{t - 3}^{d} } \right)*\left( {\Delta EPU_{t - 3} } \right)$				0.009 (0.941)
$\Delta lnX_{t}^{f}$	0.056 (0.404)	0.036 (0.608)	− 0.002 (0.974)	0.002 (0.973)
$ln\left( {X/Q} \right)_{t}^{d}$	0.067** (0.034)	0.071** (0.023)	0.064** (0.020)	0.071** (0.016)
$ln\left( {X/Q} \right)_{t}^{f}$	0.026** (0.029)	0.017 (0.234)	0.002 (0.898)	− 0.008 (0.654)
${\text{ln}}\left( {A^{JPN} /A^{LKA} } \right)_{t - 1}$	0.063 (0.115)	0.031 (0.452)	− 0.011 (0.779)	− 0.031 (0.494)
$\delta$		− 0.098 (0.668)	0.478* (0.077)	0.519* (0.075)
$R^{2}$	0.246	0.341	0.528	0.545

The table shows estimates of the TFP growth regression: $\Delta lnA_{t} = \gamma_{0} + \gamma_{1} (\Delta \ln X_{t}^{d} )*(\Delta EPU_{t} ) + \gamma_{2} (\Delta \ln X_{t - 1}^{d} )*\left( {\Delta EPU_{t - 1} } \right) + \gamma_{3} (\Delta \ln X_{t - 2}^{d} )*\left( {\Delta EPU_{t - 2} } \right) + \gamma_{4} (\Delta \ln X_{t - 3}^{d} )*\left( {\Delta EPU_{t - 3} } \right) + \gamma_{5} \Delta lnX_{t}^{f} + \gamma_{6} ln\left( \frac{X}{Q} \right)_{t}^{d} + \gamma_{7} ln\left( \frac{X}{Q} \right)_{t}^{f} + \gamma_{8} ln\left( {\frac{A}{A}_{LKA}^{JPN} } \right)_{t - 1} + e_{t} .$ In this regression model, EPU influence TFP growth ($\Delta lnA$) through domestic R&D ($X^{d}$). This impact is captured by the interaction terms between $EPU$ and $X^{d}$. We include the contemporaneous interaction term as well as lagged interaction terms up to three lags, to capture the persistent impact of EPU on R&D and TFP growth. From the second (2) to fourth (4) regressions, we estimate the joint impact of the interaction terms, and this is captured by the parameter, $\delta = \mathop \sum \limits_{i = 0} \gamma_{i + 1}$. The variables, $X^{f}$, $X/Q$, and $A^{JPN} /A^{LKA}$, denote, respectively, Japan’s R&D, Sri Lanka’s R&D intensity measured as R&D per labour, and technology frontier. Coefficients and *p-value*s are, respectively, outside and inside the parentheses

*, **, and *** Statistical significance at 10%, 5%, and 1% levels. Our sample is from 1980 to 2018

In terms of economic significance, these estimates suggest that a unit standard deviation increase in EPU would contemporaneously decrease TFP growth by − 3.43% of its sample mean (i.e. 0.79%) via a reduction in R&D investment.^16^ One and two years after the unit standard deviation increase in EPU, TFP growth increases by 4.57% and 4.20%, respectively, of its sample mean of 0.79%. It is clear that Sri Lanka is a peculiar case. The country has undergone prolonged periods of uncertainty, to the extent that its TFP growth and economic indicators have fully absorbed the impact of uncertainty. Our estimates show that, although EPU affects TFP growth, as shown by the sum of the coefficients of the interaction terms, $\delta$, the impact is not sufficiently detrimental to overturn the positive impact of R&D expenditures on TFP growth. In terms of economic significance, a unit standard deviation increase in EPU would increase TFP growth by 5.44% of its sample mean of 0.79% through R&D investment in the long term.

The negative contemporaneous impact of EPU on R&D investment and TFP growth is in line with the theoretical prediction of the real options literature, where firms are generally cautious about investing during periods of uncertainty (Sarkar 2000; Bloom et al. 2007; Iyke and Ho 2020). In this case, it appears that the government’s initial hesitation to invest in R&D in response to an increase in EPU undermines TFP growth in the country. Over time, since the government, unlike firms, is more concerned about the social benefits of R&D investments, it should internalise the EPU and increase R&D investments to stimulate TFP growth, consistent with the estimated positive impact of R&D.

In addition to the impact of EPU, we find that domestic R&D intensity, measured as R&D scaled by labour (i.e. $ln\left( {X/Q} \right)^{d}$), is an important determinant of TFP growth in Sri Lanka. This reflects the difficulty in clearly distinguishing endogenous growth theories in Sri Lanka, as observed in the preceding analysis. What can be said with certainty is that R&D is an important source of TFP growth in the country. Hence, policies aimed at fortifying R&D investment would drive the country towards a higher long-term growth path.

We examine the sensitivity of these estimates to two alternative R&D intensity indicators, namely, R&D scaled by the GDP ($RD/Y$) and R&D scaled by the product of TFP and labour ($RD/AL$). Table 5 reports the estimates. These estimates are consistent with those in Table 4. Specifically, Panel A, which shows the estimates based on $RD/Y$, suggests that EPU has a negative contemporaneous impact on TFP growth via R&D investment. For the first and second lags, the impact of EPU on TFP growth via R&D is positive and statistically significant, but this significance dissipates for the third lag (see Panel A). The joint impact of EPU on TFP growth via R&D is positive and statistically significant, as shown by the sum of coefficients of the interaction terms (i.e. $\delta$). In other words, the detrimental impact of EPU is overturned in the long term as the economy internalises the impact; hence R&D boosts long-term TFP growth despite the heightened EPU. We draw the same conclusions from the estimates in Panel B, which are based on $RD/AL$.

**Table 5:** Impact of EPU on TFP growth based on alternative R&D intensity indicators

	(1)	(2)	(3)	(4)
*Panel A: RD/Y*				
Constant	0.185* (0.057)	0.134 (0.238)	0.016 (0.893)	− 0.027 (0.839)
$\left( {\Delta lnX_{t}^{d} } \right)*\left( {\Delta EPU_{t} } \right)$	− 0.346* (0.085)	− 0.304 (0.118)	− 0.301* (0.075)	− 0.310 (0.105)
$\left( {\Delta lnX_{t - 1}^{d} } \right)*\left( {\Delta EPU_{t - 1} } \right)$		0.295** (0.042)	0.437*** (0.002)	0.450*** (0.002)
$\left( {\Delta lnX_{t - 2}^{d} } \right)*\left( {\Delta EPU_{t - 2} } \right)$			0.422*** (0.001)	0.435*** (0.002)
$\left( {\Delta lnX_{t - 3}^{d} } \right)*\left( {\Delta EPU_{t - 3} } \right)$				0.017 (0.898)
$\Delta lnX_{t}^{f}$	0.018 (0.797)	0.011 (0.883)	− 0.020 (0.746)	− 0.016 (0.801)
$ln\left( {X/Q} \right)_{t}^{d}$	0.034 (0.347)	0.042 (0.235)	0.050 (0.104)	0.054 (0.111)
$ln\left( {X/Q} \right)_{t}^{f}$	0.052* (0.069)	0.038 (0.260)	0.004 (0.903)	− 0.008 (0.831)
${\text{ln}}\left( {A^{JPN} /A^{LKA} } \right)_{t - 1}$	0.062 (0.164)	0.033 (0.483)	− 0.015 (0.734)	− 0.029 (0.571)
$\delta$		− 0.008 (0.973)	0.559** (0.045)	0.592* (0.051)
$R^{2}$	0.151	0.252	0.478	0.482
*Panel B: RD/AL*				
Constant	− 0.325* (0.092)	− 0.268 (0.250)	− 0.049 (0.834)	− 0.005 (0.987)
$\left( {\Delta lnX_{t}^{d} } \right)*\left( {\Delta EPU_{t} } \right)$	− 0.284 (0.132)	− 0.249 (0.174)	− 0.252 (0.115)	− 0.235 (0.187)
$\left( {\Delta lnX_{t - 1}^{d} } \right)*\left( {\Delta EPU_{t - 1} } \right)$		0.293** (0.046)	0.438*** (0.002)	0.442*** (0.003)
$\left( {\Delta lnX_{t - 2}^{d} } \right)*\left( {\Delta EPU_{t - 2} } \right)$			0.424*** (0.002)	0.424*** (0.003)
$\left( {\Delta lnX_{t - 3}^{d} } \right)*\left( {\Delta EPU_{t - 3} } \right)$				− 0.015 (0.905)
$\Delta lnX_{t}^{f}$	0.020 (0.772)	0.014 (0.858)	− 0.030 (0.655)	− 0.030 (0.666)
$ln\left( {X/Q} \right)_{t}^{d}$	0.020 (0.568)	0.031 (0.378)	0.043 (0.168)	0.041 (0.217)
$ln\left( {X/Q} \right)_{t}^{f}$	0.021* (0.083)	0.017 (0.240)	0.003 (0.836)	0.000 (0.988)
${\text{ln}}\left( {A^{JPN} /A^{LKA} } \right)_{t - 1}$	0.050 (0.223)	0.028 (0.523)	− 0.014 (0.725)	− 0.020 (0.665)
$\delta$		0.045 (0.852)	0.610** (0.028)	0.615** (0.047)
$R^{2}$	0.138	0.238	0.464	0.461

The table shows estimates of the TFP growth regression, which uses alternative measures of R&D intensity: $\Delta lnA_{t} = \gamma_{0} + \gamma_{1} (\Delta \ln X_{t}^{d} )*(\Delta EPU_{t} ) + \gamma_{2} (\Delta \ln X_{t - 1}^{d} )*\left( {\Delta EPU_{t - 1} } \right) + \gamma_{3} (\Delta \ln X_{t - 2}^{d} )*\left( {\Delta EPU_{t - 2} } \right) + \gamma_{4} (\Delta \ln X_{t - 3}^{d} )*\left( {\Delta EPU_{t - 3} } \right) + \gamma_{5} \Delta lnX_{t}^{f} + \gamma_{6} ln\left( \frac{X}{Q} \right)_{t}^{d} + \gamma_{7} ln\left( \frac{X}{Q} \right)_{t}^{f} + \gamma_{8} ln\left( {\frac{A}{A}_{LKA}^{JPN} } \right)_{t - 1} + e_{t} .$ In this regression model, EPU influences TFP growth ($\Delta lnA$) through domestic R&D ($X^{d}$). This impact is captured by the interaction terms between $EPU$ and $X^{d}$. We include the contemporaneous interaction term as well as lagged interaction terms up to three lags, to capture the persistent impact of EPU on R&D and TFP growth. From the second (2) to fourth (4) regressions, we estimate the joint impact of the interaction terms, and this is captured by the parameter, $\delta = \mathop \sum \limits_{i = 0} \gamma_{i + 1}$. The variables, $X^{f}$, $X/Q$, and $A^{JPN} /A^{LKA}$, denote, respectively, Japan’s R&D, Sri Lanka’s R&D intensity, and technology frontier. In Panel A, R&D intensity is proxied by R&D per GDP (*Y*), while in Panel B it is proxied by R&D per the product of TFP and labour (*AL*). Coefficients and *p-value*s are, respectively, outside and inside the parentheses

*, **, and *** statistical significance at 10%, 5%, and 1% levels. Our sample is from 1980 to 2018

It is worth noting that domestic R&D intensity does not appear to significantly determine TFP growth using $RD/Y$ and $RD/AL$ as indicators of R&D intensity.^17^ In other words, we are better able to validate the semi-endogenous growth theory using these alternative R&D intensity indicators. Overall, our finding that EPU influences the impact of R&D investment on TFP growth is not driven by the measure of R&D intensity.

## Concluding remarks

This paper presents evidence in support of semi-endogenous growth theories for Sri Lanka. Using a historical time series dataset (1980–2018), it shows that Sri Lanka’s growth experience is best explained by investment in R&D and innovation. We test the growth theories using nonlinear time series frameworks that allow us to model structural changes or regime shifts in the economy. We also test our hypothesis that EPU is detrimental to TFP growth in the country and find evidence in support of this hypothesis. Specifically, we find that EPU impedes TFP growth contemporaneously via the R&D channel. Following heightened EPU, economic agents cut back on investments, including R&D investments, which leads to a reduction in TFP growth. In addition, since we demonstrate that TFP contributes more than capital and labour to economic growth in the country, economic uncertainty impedes the country’s path to success. We show that, when EPU is persistent, as in Sri Lanka’s case, the economy internalises it and, in turn, investment in R&D takes place, spurring TFP growth. Our findings imply that investment in R&D would push the country to a higher growth path and should be pursued as an active macroeconomic policy. This implication can be extended to countries with similar economic fundamentals as Sri Lanka. Since our findings suggest that the negative uncertainty effects are at best temporary, the country is better positioned to absorb future negative shocks or increases in policy uncertainty.

## Data Availability

All data used in this paper are available upon request from the authors.

## Appendix

See Table

**Table 6:** Definitions and sources of the variables

Variable	Definition/Full name	Source
Y	Gross domestic product. For Sri Lanka, this is GDP at constant prices (Rs. billion, 2010) and for Japan, this is GDP at constant 2011 national prices (in mil. 2011US$)	Sri Lanka: IMF WEO, GDP Constant Prices, Rs. Billion, 2010 Japan: PWT9.0
L	Labour force. Sri Lanka’s labour force. For Japan, this is the number of persons engaged (in millions)	Sri Lanka: World Bank, WDI and CBSL, Labor force total Japan: PWT9.0
RD	Research and development (R&D). Sri Lanka’s R&D is computed by scaling R&D (% of GDP) by 100 and multiplying the result by nominal GDP (Rs. Million). For Japan, this is R&D expenditure, in million US dollars	Sri Lanka: World Bank, WDI Research and development expenditure (% of GDP) Japan: OECD, https://data.oecd.org/rd/gross-domestic-spending-on-r-d.htm
A	This is the total factor productivity (TFP) measured in constant national prices (2011 = 100) for Sri Lanka and Japan	Sri Lanka: FRED Japan: PWT9.0
PATENT	Number of Patent Applications	Sri Lanka: WIPO and NIPO Japan: World Development Indicators
EPU	Economic policy uncertainty. This is the news-based World Uncertainty Index for Sri Lanka, constructed by Ahir et al. (2018)	https://www.policyuncertainty.com/wui_quarterly.html

The table presents the definitions/full names and sources of the variables used in the empirical analysis

6.

## Footnotes

1 See https://www.scmp.com/economy/china-economy/article/3146489/us-china-trade-war-timeline-key-dates-and-events-july-2018.

2 See https://commonslibrary.parliament.uk/research-briefings/cbp-7960/.

3 See https://www.reuters.com/article/us-hongkong-protests-timeline-idUSKBN23608O.

4 See https://www.businessinsider.com.au/oil-price-crash-market-drop-global-price-war-futures-coronavirus-2020-3?r=US&IR=T.

5 Central Bank of Sri Lanka, Annual Reports, various years.

6 EPU is an economic risk, which describes states, whereby government and monetary policies, electoral outcomes, political regimes, tax regimes, among others, are uncertain, causing negative business sentiments, a rise in risk premia, and delayed investment and spending (see Ahir et al. 2018).

7 Central Bank of Sri Lanka, Annual Reports, various years.

8 Central Bank of Sri Lanka, Annual Reports, various years.

9 Central Bank of Sri Lanka, Annual Reports, various years.

10 Madsen et al. (2010) model nonlinearity in the relationship between TFP growth and its determinants exogenously by utilizing pre-reform and full samples of data.

11 This database can be accessed at https://www.rug.nl/ggdc/productivity/pwt/pwt-releases/pwt9.0?lang=en.

12 Japan is the most developed country in Asia and hence serves as the most appropriate benchmark for assessing Sri Lanka’s technological progress.

13 We use the exponential growth function ${y}_{t}=\alpha {k}^{t}$, where $t$ is the time period, ${y}_{t}$ is the variable with missing observations, and $k$ is a constant base for $t$. This forecasting model is more flexible as it allows for both linear and nonlinear patterns in the data. Note that we considered a linear trend fit, but the forecast values were unreasonably large.

14 This index can be accessed at https://www.policyuncertainty.com/wui_quarterly.html.

15 Prior studies observe this nonlinear behaviour and address it. Madsen et al. (2010) overcome this limitation by estimating the models using pre- and post-reform subsamples. However, their approach fails to explicitly model the intrinsic structural changes. Juhro et al. (2020) address this issue by explicitly modelling the structural changes, using nonlinear cointegration techniques. Our study is inspired by both studies but follows the latter when addressing the underlying nonlinearities.

16 The measure of the EPU index is based on the number of words such as *uncertain*, *uncertainty*, and *uncertainties* that appear in the country Economist Intelligence Unit reports each quarter, scaled by the total number of words in each report (Ahir et al. 2018). Hence, a unit standard deviation increase in the EPU in Sri Lanka (i.e. 0.08) cannot be meaningfully interpreted, since the index is not measured in any standard unit.

17 This is consistent with Hünermund and Czarnitzki (2019), who found that R&D grants have no average effect on growth at the firm level.
